# Relationship between Cerebrospinal Fluid Biomarkers for Inflammation, Demyelination and Neurodegeneration in Acute Optic Neuritis

**DOI:** 10.1371/journal.pone.0077163

**Published:** 2013-10-08

**Authors:** Signe Modvig, Matilda Degn, Henrik Horwitz, Stig P. Cramer, Henrik B. W. Larsson, Benedikte Wanscher, Finn Sellebjerg, Jette L. Frederiksen

**Affiliations:** 1 MS Clinic, Department of Neurology, Copenhagen University Hospital Glostrup, Glostrup, Denmark; 2 Functional Imaging Unit, Department of Diagnostics, Copenhagen University Hospital, Glostrup, Glostrup, Denmark; 3 Department of Clinical Neurophysiology, Copenhagen University Hospital Glostrup, Glostrup, Denmark; 4 Danish MS Research Centre, Department of Neurology, Copenhagen University Hospital Rigshospitalet, Copenhagen, Denmark; Innsbruck Medical University, Austria

## Abstract

**Background:**

Various inflammatory biomarkers show prognostic potential for multiple sclerosis (MS)-risk after clinically isolated syndromes. However, biomarkers are often examined singly and their interrelation and precise aspects of their associated pathological processes remain unclear. Clarification of these relationships could aid the appropriate implementation of prognostic biomarkers in clinical practice.

**Objective:**

To investigate the interrelation between biomarkers of inflammation, demyelination and neurodegeneration in acute optic neuritis and to assess their association to measures of MS risk.

**Material and Methods:**

A prospective study at a tertiary referral centre from June 2011 to December 2012 of 56 patients with optic neuritis as a first demyelinating symptom and 27 healthy volunteers. Lumbar puncture was performed within 28 (median 16) days of onset. CSF levels of CXCL13, matrix metalloproteinase (MMP)-9, CXCL10, CCL-2, osteopontin and chitinase-3-like-1, myelin basic protein (MBP) and neurofilament light-chain (NF-L) were determined. MS-risk outcome measures were dissemination in space (DIS) of white matter lesions on cerebral MRI, CSF oligoclonal bands and elevated IgG-index.

**Results:**

In the interrelation analysis the biomarkers showed close correlations within two distinct groups: Biomarkers of leukocyte infiltration (CXCL13, MMP-9 and CXCL10) were strongly associated (p<0.0001 for all). Osteopontin and chitinase-3-like-1 were also tightly associated (p<0.0001) and correlated strongly to tissue damage markers (NF-L and MBP). The biomarkers of leukocyte infiltration all associated strongly with MS-risk parameters, whereas CHI3L1 and MBP correlated with MRI DIS, but not with CSF MS-risk parameters and osteopontin and NF-L did not correlate with any MS-risk parameters.

**Conclusions:**

Our findings suggest two distinct inflammatory processes: one of leukocyte infiltration, represented by CXCL13, CXCL10 and MMP-9, strongly associated with and potentially predicting MS-risk; the other represented by osteopontin and CHI3L1, suggesting tissue damage-related inflammation, potentially predicting residual disabilities after attack and perhaps cumulative damage over time. These hypotheses should be further addressed in follow-up studies.

## Introduction

Optic neuritis (ON) is an inflammatory condition in the optic nerve causing blurred vision and retroorbital pain, usually remitting partially or completely within months after onset. Often ON is idiopathic in origin, but around half of patients with ON and no prior neurological symptoms will go on to develop multiple sclerosis (MS) [1]. MS is an immune-mediated, demyelinating disease of the central nervous system (CNS), where activated immune cells are thought to cross the blood-brain barrier (BBB) and orchestrate a broad immunological reaction towards myelin components, leading to demyelination and subsequent axonal damage. Thus MS has both inflammatory and neurodegenerative features. How inflammation and neurodegeneration interact is, however, a matter of ongoing debate [2]. Especially in the earliest stages of MS, the contribution of these processes to the future course of disease remains unclear [3].

The clinical use of cerebrospinal fluid (CSF) biomarkers in predicting the development of MS after a first demyelinating event, a clinically isolated syndrome (CIS) such as ON, has for many years been restricted to the assessment of intrathecal IgG-synthesis (IgG index and in particular oligoclonal bands (OCB)) [4,5]. However, many other CSF biomarkers have shown potential in the field [6-8]. In this study, we focus on biomarkers of leukocyte infiltration (CXCL13, MMP-9, CXCL10 and CCL2), inflammation (CHI3l1 and OPN) and tissue damage (NF-L and MBP).

The aim of the study is to clarify the interrelation between these markers, and their relationship with the features of the ON itself, i.e. severity and temporal development, and to established predictors of MS risk in CSF and on magnetic resonance imaging (MRI). We prospectively studied a group of patients with ON and no prior demyelinating symptoms, all sampled within a narrow time window from symptom onset. This provided the uniformity in symptoms and temporal development of disease necessary to make valid assessments of the factors associated with the biomarker levels during ON as a first demyelinating event.

## Material and Methods

### Patients

Fifty-six patients with acute ON were enrolled prospectively in the study. From June 2011 to December 2012 132 patients were referred by ophthalmologists to the Clinic of Optic Neuritis, Glostrup Hospital with suspected ON. Inclusion criteria were as follows: A diagnosis of ON based on the below defined criteria; time from onset of visual symptoms to lumbar puncture of ≤ 28 days; age between 18 and 59 years (both included); and no prior symptoms of demyelinating disease (a prior episode of unspecific sensory symptoms with no objective equivalent at clinical examination or somatosensory evoked potential testing was allowed).

Thirty-six patients were excluded as they did not meet the inclusion criteria of diagnosis (n=15), age (n=3) or duration of symptoms (n=18). Thirteen patients had previous MS symptoms and 12 patients underwent lumbar puncture elsewhere. Five had received glucocorticosteroids prior to lumbar puncture (two for ON, three for other causes). The symptoms of three patients were found to have another organic cause (sarcoma of the lateral rectus muscle (1), sarcoidosis (1), central serous chorioretinopathy (1)). Seven patients were eligible but declined to participate after the informed consent procedure. Thus, 56 (89%) of 63 eligible patients participated in the study. All included patients underwent the full diagnostic program.

The diagnosis of ON was verified by the presence of a classical symptomatology and disease course (blurred vision, accompanied by retrobulbar pain often accentuated by eye movement, worsening over hours to a few days) and no other organic cause of their symptoms, along with a minimum of three out of the following four objective findings: decreased visual acuity/low contrast visual acuity, a relative afferent pupillary defect, dyschromatopsia upon formal testing, or a visual field defect upon automated perimetry. If patients had early remission upon examination, data from the referring ophthalmologist were also accepted for fulfilment of the inclusion criteria.

### Healthy controls

Twenty-seven healthy controls (HC) were selected from the general population on the basis of age and gender through a web portal for research study volunteers. None had a history of autoimmune, neurological or chronic illness, nor any prior symptoms indicating demyelinating disease or any family members with MS. All underwent a thorough clinical and neurological examination. Eleven HC additionally underwent a cerebral 3 Tesla MRI as part of a related project. None had white matter lesions. Demographic data of patients and HC are shown in table 1.

**Table 1:** Demographic and paraclinical data of patients and healthy controls.

**Data**	**Healthy controls**	**Patients**	**P-value**
N	27	56	
Female:male ratio	21:6	43:13	0.92 (Mann-Whitney)
Age	33 y (26-43 y)	36 y (29-46 y)	0.23 (Mann-Whitney)
CSF leukocyte count	2 (2-4, ref <5 x 10^3^/ml)	5 (3-12, ref < 5 x 10^3^/ml)	
CSF protein	0.29 (0.20-0.40, ref 0.15-0.50 g/l)	0.29 (0.16-0.43, ref 0.15-0.50 g/l)	
Albumin quotient (CSF/serum)	0.0042 (0.0033-0.0053, ref < 0.0068)	0.0048 (0.0039-0.0068, ref < 0.0068)	
IgG index	0.49 (0.47-0.52, ref < 0.70)	0.61 (0.50-0.91, ref < 0.70)	
Oligoclonal band positive	0/27 controls	30/56 patients (54%)	
Visual acuity Snellen/ETDRS	N/A	0.5 (0.0125-0.8)/51 (2-64) letters	
Low contrast sensitivity loss at 5%	N/A	43 (22-58) letters	
Low contrast sensitivity loss at 1.25%	N/A	35 (27-45) letters	
VEP latency at 9mm checkerboards	N/A	145 (122.5-250*) ms	
Time from onset to lumbar puncture	N/A	16 (14-20) days	
MRI T2 lesions**	0 (0-0)	2 (0-9)	
Dissemination in space on MRI	0/11 HC	26/56 patients (46%)	
Gd-enhancing lesions	0 (0/11 HC)	14 lesions (9/53*** patients)	
Optic neuritis with no MRI or CSF abnormalities	N/A	10/56 patients (18%)	

All continuous values represent the median (interquartile range)

* Latency was set to 250 ms (= max detection limit) when there was no cortical response

** Unspecific subcortical lesions not included

*** 3 patients did not receive Gd-contrast

### Clinical examination and visual tests of patients

Visual acuity was tested by Early Treatment of Diabetic Retinopathy Score charts and low contrast visual acuity was assessed by Low Contrast Sloan Letter charts at 5% and 1.25%. Visual evoked potentials (VEP) were performed with 9 mm, and upon indication 4.5 and 18 mm, checkerboards, assessing latency and amplitude of the visual pathway conduction signal on either eye. Furthermore, a full clinical and neurological examination was performed.

### MRI

The patients underwent MRI of the brain and cervical column on a Philips Achieva 3 Tesla system with 0.1 mmol/kg body weight Gadovist (Gd) enhancement studies. The presence and number of white matter lesions were assessed on the axial T2-weighted fluid attenuated inversion recovery (FLAIR) sequence (35 slices, echo time (TE)=125 ms, repetition time (TR)=11000 ms, field of view (FOV)=230x119, Matrix size=352x231, slice thickness of 3.5 mm) and Gd-enhancement was assessed on the post-contrast axial T1-weighted sequence (44 slices, TE=60 ms, TR=600 ms, FOV=240x132, Matrix size=256x192). The 11 HC, who underwent MRI, were scanned by the same protocol. Two patients were due to overweight resp. claustrophobia scanned in a 1.5 Tesla open MRI scanner. Dissemination in space of MRI white matter lesions was defined as a minimum of one T2 hyperintense lesion of > 3mm in at least two of the following four areas of the CNS: periventricular, juxtacortical, infratentorial or spinal cord. Gd enhancement of the affected optic nerve was not included in the registration of Gd enhancing lesions.

### CSF and blood tests

All patients and HC underwent a lumbar puncture with analysis of CSF leukocyte and erythrocyte counts, CSF protein and glucose levels, IgG index and albumin quotient and test for OCB by isoelectric focusing and immunoblotting, as well as a broad panel of serological tests. For the patients this included tests of other known inflammatory, autoimmune and infectious causes of ON. Paraclinical characteristics of the patients and HC are shown in table 1.

### CSF sampling and biomarker analysis

12 ml of CSF was collected in an ice bath and immediately centrifuged at 400G at 4°C for 10 min, and the cell-free supernatant was frozen and stored in 0.5 ml aliquots at -80°C until analysis. All biomarker analyses were performed by enzyme-linked immunosorbent assays (ELISA) using commercially available kits. CXCL13, CXCL10, CCL2, MMP-9, CHI3L1 and OPN from R&D systems, Abingdon, UK (DCX130, DMP900, DIP100, DCP00, DOST00 and DC3L10), MBP from Beckman & Coulter, US (DSL-10-58300) and NF-L from UmanDiagnostics AB, Sweden. All assays were conducted according to the manufacturer’s recommendations and dilutions were made for OPN (1:50), CCL2 (1:1), CXCL10 (1:5), NFL (1:1) and CHI3L1 (1:200) to fit the optimal detection range. For MMP-9 and CXCL13, some values were below the minimal detection limits of the assays. For NF-L, this was the case for one HC. Values were then assigned corresponding to the lowest concentration of the standard curves of the analyses (CXCL13: 3.9 pg/ml, MMP-9: 15.6 ng/ml, NF-L: 100ng/l). All samples were run in duplicate, all plates included both patients and HC and were analysed blinded to the clinical status of the samples. Mean intra-assay variation was 2.2% for CXCL13, 2.3% for MMP-9, 2.6% for CXCL10, 2.3% for CHI3L1, 3.5% for OPN, 2.0% for NF-L, 2.0% for MBP and 2.1% for CCL-2. Only results with a duplicate variation of less than 7.5% from the duplicate mean were accepted. Mean inter-assay variation was 15.6% for CXCL13, 7.2% for MMP-9, 1.3% for CXCL10, 3.9% for CHI3L1, 10.1% for OPN, 10.6% for NF-L, 4.9% for MBP and 0.6% for CCL-2 and was calculated from an internal CSF quality control present on all plates. Standard curves were fitted by a 5-parameter logistic regression model.

### Statistical methods

All statistical analyses were performed in SAS 9.2. Due to small numbers, non-parametric methods were used for comparison between groups (Mann-Whitney U test) and for correlation analysis (Spearman’s rank correlation). Multiple linear regression analysis was used to test the influence of age and gender in HC and to control for age in the interrelation analysis of the biomarkers. A censored regression (tobit) model was used for CXCL13 and MMP-9. The results of the biomarker correlation analyses were Bonferroni corrected by a factor of 28 and results of the comparison to parameters of MS-risk and ON severity and timeframe were Bonferroni corrected by a factor 8. Results with a p-value < 0.05 after this correction were considered significant. All p-values reported in the results section are the Bonferroni corrected p-values.

### Ethics

All individuals participated voluntarily in the study after a thorough oral and written information procedure. Oral and written consent were obtained from all participants. The study including the informed consent procedure was approved by the regional scientific ethics committee of Copenhagen, Denmark (protocol H1-2011-019).

## Results

### Biomarker levels in patients and healthy controls

CSF biomarker levels of the patients and HC are shown in table 2. Levels of CXCL13 (p<0.0001), MMP-9 (p<0.0001), CXCL10 (p=0.0018), NF-L (p<0.0001), MBP (p=0.0229) and CHI3L1 (p=0.0084) were significantly higher in patients, whereas there was no significant difference between the groups for OPN (p=0.1448) or CCL2 (p=0.5416). None of the 27 HC resp. 33/56 patients had detectable levels of CXCL13. MMP9 was detectable in only 1 HC at a concentration of 18.9 pg/ml, whereas 31/56 patients had detectable levels of MMP-9. OPN (p<0.0001), NF-L (p<0.0001), CHI3L1 (p=0.0073) and MBP (p=0.0022) were found to increase significantly with age in HC. None of the biomarkers differed between men and women in HC.

**Table 2:** Biomarker levels of patients and healthy controls.

	**Healthy controls**			**Patients**			
	**Median**	**IQR**	**Range**	**Median**	**IQR**	**Range**	**P-value**
**CXCL13 (pg/ml)**	3.9	3.9-3.9	3.9-3.9	6.9	3.9-30.3	3.9-495.8	P<0.0001
**MMP-9 (ng/ml)**	0.156	0.156-0.156	0.156-0.189	0.166	0.156-0.459	0.156-5.846	P<0.0001
**CXCL10 (pg/ml)**	109.7	76.5-133.3	46.5-315.1	176.8	110.1-274.0	44.3-1120.9	P=0.0018
**CHI3L1 (ng/ml)**	71.1	51.1-98.2	31.6-175.6	95.4	71.2-136.7	18.1-466.6	P=0.0084
**OPN (ng/ml)**	127.2	106.3-160.7	75.3-235.7	146.5	117.1-227.9	46.1-530.0	P=0.1448
**NF-L (ng/l)**	414.1	261.9-629.6	100.0-853.2	1476.3	1024.4-3035.9	267.4-10112.8	P<0.0001
**MBP (ng/ml)**	0.714	0.526-0.840	0.350-1.045	0.818	0.652-1.071	0.432-4.813	P=0.0229
**CCL2 (pg/ml)**	417.1	330.7-468.6	299.5-680.8	441.7	358.3-503.9	147.6-679.4	P=0.5416

Biomarker levels in HC and patients with acute ON. Levels differed significantly for CXCL13, MMP-9, CXCL10, CHI3L1 and NF-L. Testing was done by Mann-Whitney U test. IQR: interquartile range.

### Interrelation of biomarker levels in patients with optic neuritis

The correlation between the biomarkers is shown in Table 3. A close correlation was seen between CXCL13, MMP9 and CXCL10 (p<0.0001 for all). Likewise, close correlations were seen between CHI3L1, OPN, MBP and NF-L. As the latter four markers were all shown to increase with age in HC, the estimates were corrected for age differences in multivariate linear regression analyses. All significant correlations remained so in these analyses.

**Table 3:** Interrelation of CSF biomarker levels in patients with acute ON.

	**CXCL13**	**MMP-9**	**CXCL10**	**CCL2**	**CHI3L1**	**OPN**	**NF-L**	**MBP**
**CXCL13**								
**MMP-9**	**r= 0.91 (p<0.0001)**							
**CXCL10**	**r=0.64 (p<0.0001)**	**r=0.73 (p<0.0001)**						
**CCL2**	r=-0.32 (p=0.52)	r=-0.28 (p=1)	r=-0.03 (p=1)					
**CHI3L1**	r=0.12 (p=1)	**r=0.22 (p=1)**	**r=0.37 (p=0.1428)**	r=0.38 (p=0.1563)				
**OPN**	r=0.14 (p=1)	r=0.17 (p=1)	r=0.36 (p=0.1661)	r=0.21 (p=1)	**r=0.76 (p<0.0001)**			
**NF-L**	r=0.35 (p=0.2352)	r=0.32 (p=0.4411)	r=0.30 (p=0.6872)	r=-0.03 (p=1)	r=0.39 (p=0.0730)	**r=0.50 (p=0.0023)**		
**MBP**	r=0.29 (p=0.8722)	r=0.34 (p=0.2685)	r=0.36 (p=0.1650)	r=-0.02 (p=1)	**r=0.65 (p<0.0001)**	**r=0.54 (p=0.0005)**	**r=0.61 (p<0.0001)**	

The correlations between CSF biomarker levels are shown. The biomarkers are seen to correlate strongly within two groups: CXCL13, CXCL10 and MMP-9 resp. CHI3L1, OPN, NF-L and MBP. Statistical testing was done by Spearman’s rank correlation analysis. All listed p-values have been Bonferroni corrected by a factor 28. Correlations in bold letters: significant after age-adjustment in multiple linear regression analyses. R=Spearman’s rho.

### Biomarkers, measures of ON and established MS risk factors

Levels of the individual biomarkers were compared to measures related to ON, representing severity and time course (time from onset to CSF sampling, subjective remission at time of CSF sampling and a combined measure of visual acuity and low contrast visual acuity), as well as measures related to MS risk (presence of OCB in the CSF, dissemination in space on MRI, Gd-enhancing lesions on MRI, CSF leukocyte count and IgG-index). Furthermore, the potential influence of the CSF/serum albumin quotient was examined (data not shown).

**Figure 1:**
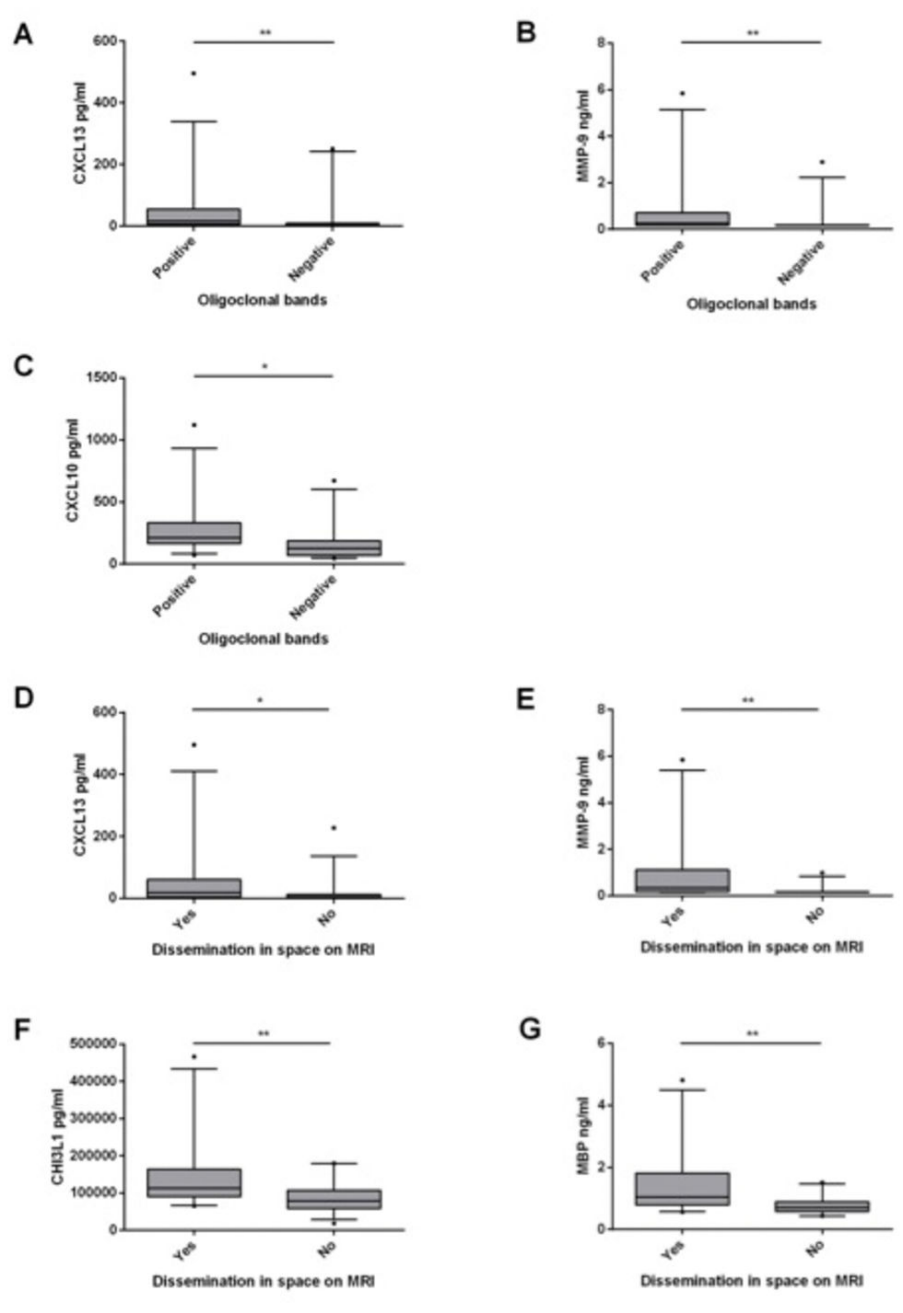
Biomarker levels, CSF oligoclonal bands and MRI dissemination in space in patients with acute ON. Panel A-C: The association between biomarker levels and the presence of OCB in the CSF. Panel D-G: The association between biomarker levels and dissemination in space on MRI, defined as a minimum of one T2 hyperintense white matter lesion of > 3mm in at least two of the following four areas of the CNS: periventricular, juxtacortical, infratentorial or spinal cord. All p-values are Bonferroni corrected by a factor 8 and only associations significant after Bonferroni correction are shown. Line: Median Box: Interquartile range. Whiskers: 5-95 percentiles. R: Spearman’s rho. *: P<0.05. **: P<0.01. ***: P<0.001.

CXCL13, CXCL10 and MMP-9 correlated with the IgG index and CSF leukocyte count (IgG index: r=0.73 for CXCL13; r=0.59 for CXCL10; and r=0.75 for MMP-9. CSF leukocyte count: r=0.57 for CXCL13; r=0.62 for CXCL10; r=0.66 for MMP-9. All p<0.0001) and with the presence of OCB (p=0.0040 for CXCL13, p=0.0128 for CXCL10 and p=0.0048 for MMP-9). A significant association was also seen with dissemination in space on MRI for MMP-9 (p=0.0024) and CXCL13 (p=0.0256) (Figure 1). For CXCL10, there was a trend that did not reach statistical significance after adjusting for multiple comparisons (p=0.0656).

**Figure 2:**
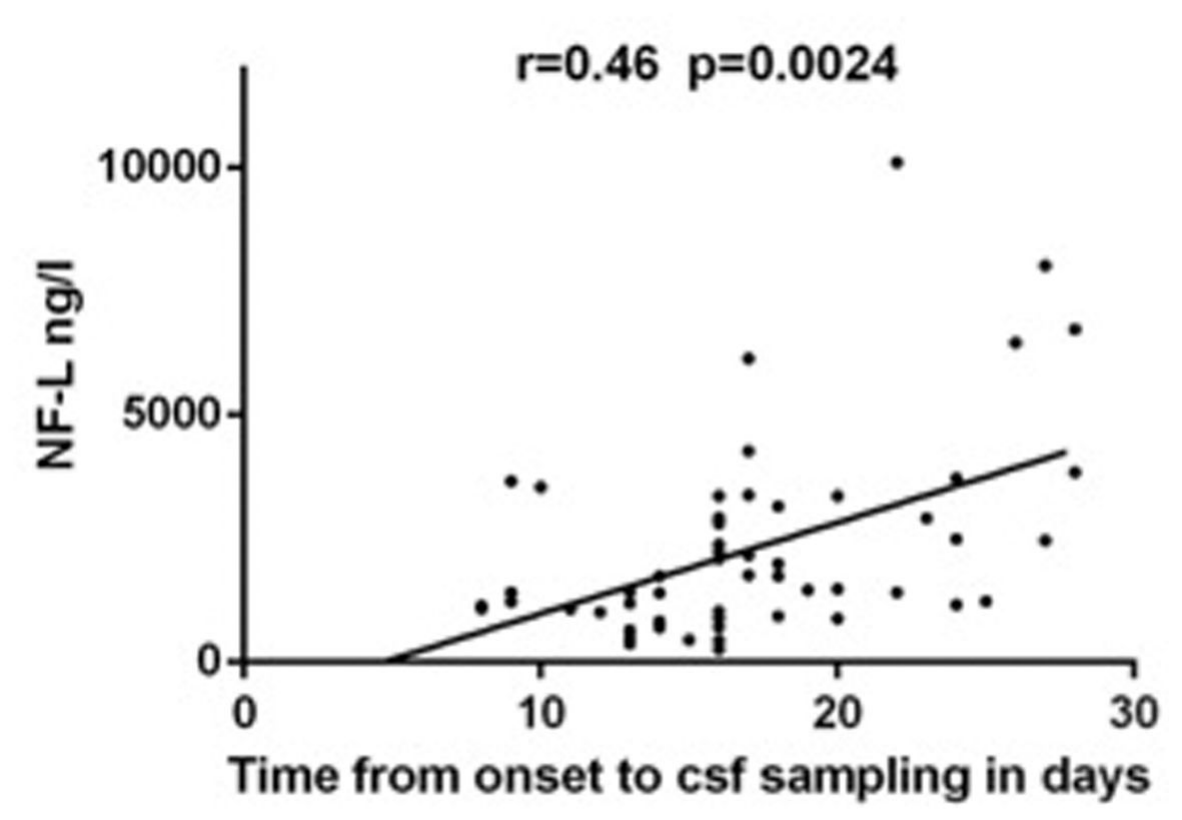
Correlation between NF-L and time from onset to CSF sampling in patients with acute ON. NF-L levels in the CSF of patients with ON is seen to correlate positively to time from onset of symptoms to CSF sampling. P-value is Bonferroni corrected by a factor 8. R: Spearman’s rho.

Furthermore, CHI3L1 (p=0.0096) and MBP (p=0.0016) were associated with the dissemination in space on MRI (Figure 1) but not with any of the CSF parameters. For NF-L, there was a trend towards significance for dissemination in space (p=0.0512). Only NF-L correlated to the time from onset of symptoms to CSF sampling, showing increased levels of NF-L with increasing time from onset to sampling (r=0.46, p=0.0024, Figure 2). OPN and CCL2 did not correlate significantly with any of the parameters examined.

None of the biomarkers correlated with the severity of visual symptoms, subjective remission or Gd-enhancing lesions on MRI (data not shown).

To take the non-measurable levels of CXCL13 and MMP-9 further into account, these were set to 0. The optimal cut-off levels of both markers were calculated using the Youden-index and were 0 for both markers. Subsequent categorical testing against the other biomarkers, clinical and paraclinical parameters still showed highly significant relations between CXCL13, MMP-9 and CXCL10 (p<0.0001 for all) and between CXCL13, MMP-9 and all CSF MS-risk parameters (p<0.0001 for all). For MMP-9 the association to dissemination in space on MRI remained significant (p=0.02) after correcting for multiple comparisons but was lost for CXCL13 (p=0.0864) after correcting for multiple comparisons.

## Discussion

In this study we have examined biomarkers of inflammation, demyelination and neurodegeneration in acute ON. We studied the relationship between the biomarkers and found them to be closely correlated in two distinct groups with much weaker between-group correlations. Interestingly, the relationship of the inflammatory biomarkers to features of MS risk, age and markers of tissue damage also showed several within-group similarities. This suggests that they could in fact represent two different aspects of the inflammatory process providing distinct prognostic information.

The first of these biomarker groups consisted of the chemokines CXCL13 and CXCL10 along with MMP-9. They can be considered biomarkers of trafficking of activated leukocytes into the subarachnoid and perivascular space [9]. In line with this, we found them closely correlated with all of the established parameters for MS risk after ON in CSF and on MRI. In contrast, the markers were not associated with the severity of ON, nor to markers of demyelination or neurodegeneration.

MMP-9 is involved in the breakdown of the basal membrane of the BBB by activated mononuclear cells [10] and CXCL13 attracts B-cells to the CNS. CXCL10 is expressed by monocytes, endothelial cells and astrocytes and the receptor for CXCL10 is upregulated upon activation of T-cells, and CXCL10 thus attracts activated cells to the CNS [11]. The infiltration of activated, autoreactive T-cells across the BBB into the CNS is a process believed by many to be the initiating event in MS pathology. Accordingly, biomarkers representing this infiltration during ON are likely to be of prognostic value regarding the later development of MS. Such a role has indeed been suggested for CXCL13 [7,12]. Furthermore, CXCL13 is known to be associated with the formation of ectopic lymphoid follicles in the meninges [13], a feature of secondary progressive disease, and it is possible that all three markers represent a meningeal component of inflammation, recently shown to be present already in early MS [14].

The second group of biomarkers consists of the two inflammatory markers CHI3L1 and OPN, along with markers of tissue damage, NF-L and MBP. These four markers correlated strongly to each other, suggesting that CHI3L1 and OPN levels could represent the component of inflammation determining the degree of tissue damage during an attack of ON.

CHI3l1 is a chitin-binding protein lacking the enzymatic activity of the true chitinases. CHI3L1 is known to play a role in cancer and chronic inflammation [15]. In rheumatoid arthritis CHI3L1 serum levels are elevated and correlate with disease activity and progression [16]. CSF levels of CHI3L1 have only been examined in CIS/RRMS in a few studies [8,17,18], where one study has suggested it as a prognostic marker for the conversion to CDMS [8]. CHI3L1 is expressed in macrophages and endothelial cells and also in astrocytes in MS and other neurological conditions [17], although its precise role in MS inflammation is still to be determined.

In spite of the close association to CHI3l1 and tissue damage markers, OPN levels were not elevated in patients compared to HC and showed no association to MS-risk parameters. OPN is a pleiotropic protein involved in bone remodelling, cancer and inflammation, and as such it is not specific for demyelinating pathology. However, OPN has shown importance for the relapse frequency and disease severity in experimental autoimmune encephalomyelitis [19], and has been linked to the diminished apoptosis of activated T-cells [20], suggesting a role for OPN in the relapsing and progressive course of MS over time. This could explain our findings, since the role of OPN might increase with disease duration. In line with this, CSF OPN levels has previously been found elevated in CSF and plasma in MS [21-24], however, only one study reported slightly elevated CSF levels in CIS patients [21]. Alternatively, an attack-related rise in OPN could happen prior to symptoms, as has been suggested from a serial study of OPN in serum [25]. A very recent paper examining a large group of MS-patients in different stages showed a negative correlation between CXCL13, MMP-9, NF-L and OPN with age, especially in patients above 54 years of age, suggesting that the inflammation subsides. They furthermore found a close correlation between CXCL13 and MMP-9 in their younger patients and weaker correlations to OPN and NF-L [26] consistent with our findings,

NF-L levels increased with time from onset to CSF sampling, suggesting a delayed axonal degeneration after demyelination. This is in line with a sequential study on NF-L [27], as well as an earlier study showing a correlation between early levels of MBP and subsequent levels of NF heavy chain after a demyelinating event [28]. It is thus possible that NF-L levels measured later in the ON course would be more representative of the degree of axonal damage, perhaps showing even stronger correlations with OPN, MBP and CHI3L1. Axonal damage has been proven present already in CIS and some studies have shown an association between severity of the attack and other neurodegenerative markers [29]. We did not see an association between NF-L and the visual function in the acute stage of disease, which might be due to this suspected delayed rise in the CSF levels of the marker. CSF levels of NF-L have been shown to correlate with EDSS in MS [6,30,31]. One study also included CIS patients [6], however, they did not adjust for age and in a recent study of NF-L in CIS the correlation between NF-L and EDSS was lost after age-adjustment [32]. Others also failed to show a correlation to EDSS in CIS [27], also attributing their finding to a delayed rise in NF-L levels.

MBP, previously shown to be weakly correlated with the severity of ON [33], did not associate to the ON severity or temporal course in our material, but was strongly associated with dissemination in space on MRI. CSF levels of MBP could represent both the damage from the ongoing attack and a more widespread breakdown of myelin, including that of subclinical lesions on MRI, providing one explanation for the lack of association to severity of the acute optic nerve demyelination. Also, anatomical factors such as the distance from the optic nerve to the lumbar CSF sampling site [34] could be of importance to our observed lack of association between tissue damage markers and ON severity, especially since the average visual acuity of our patient group is slightly higher, thus suggesting milder cases than in the study showing a correlation between MBP and visual function [33].

None of the biomarkers of the second group (OPN, CHI3L1, NF-L or MBP) showed correlations to CSF OCB, IgG index or CSF leukocyte count, supporting the notion that they associate to a different and perhaps more parenchymatously distributed inflammatory reaction than the biomarkers of leukocyte infiltration.

We cannot from this study exclude early attack-related peaks in some biomarkers, as this could have happened even prior to our sampling, though this was done very shortly after onset. When looking at biomarkers with prognostic potential, however, it is of value that sampling conditions represent those feasible in clinical settings.

Our ON patient group appears representative in terms of MS-risk. Forty six percent of the included patients presented with dissemination in space on MRI and 54% with OCB (Table 1). The frequency of OCB is slightly lower than previously reported in CIS [4,35]; however, the percentage of patients with no MRI or CSF abnormalities (18%) is in line with previous studies of ON [36].

The influence of the albumin quotient was tested and we found associations for CXCL10 and CHI3L1 (r=0.34, p=0.0109, resp. r=0.27, p=0.0448 uncorrected). For both, however, CSF levels have been shown to be higher than serum levels [8,18,37], making it unlikely that peripheral concentrations should contribute to our results.

In conclusion, our findings suggest two separate but parallel inflammatory processes occurring during ON as a first demyelinating event: one possibly relating to future risk of MS, the other associated with the degree of tissue damage. Thus, CXCL13, MMP-9 and CXCL10 could potentially predict the risk of developing MS after ON, although long-term studies of the conversion rate are needed to substantiate this. In contrast, CHI3L1 and OPN correlated with markers of tissue damage and could potentially hold prognostic value for the extent of recovery in ON and possibly for the disability progression in MS after repeated relapses. This would need to be addressed, however, in studies of ON remission and of subsequent MS-related disability and prognosis.
